# Supplementary material for: Experiences on the implementation and maintenance of the Canadian Disability Participation Project: A mixed-methods study
Source: PLoS One. 2025 Nov 13;20(11):e0334835. doi: 10.1371/journal.pone.0334835 (PMC12614619; doi:10.1371/journal.pone.0334835)
Supplement: S1 Table — (DOCX) [file pone.0334835.s001.docx]

**S1 Table.** **Team members’ roles and positionality statements**

| **Name** | **Organization** | **Roles** | **Author information** |
| --- | --- | --- | --- |
| Femke Hoekstra (she/her/hers) | University of British Columbia | Researcher | FH is an Assistant Professor UBC’s Department of Medicine and started in January 2024. Prior to her position, FH was a postdoctoral researcher who worked under the supervision of HG and KMG at the UBC. FH is a white, cis-gender, straight, able-bodied woman, who lives and works on the traditional and unceded territory of the Syilx Okanagan Nation.  Her research focuses on health services and care for equity-deserving groups in rural, remote and other isolated communities. My research involves studying implementation processes of health innovations (e.g. interventions, technologies, apps) in real-world settings from different perspectives (organization, professional, client/patient). |
| Alanna Shwed | University of British Columbia | Researcher  Trainee | AS is a PhD Candidate who works under the supervision of HG at the University of British Columbia Okanagan. AS identifies as a white, cis-gendered, able-bodied woman of settler ancestry living and working on the traditional and unceded territory of the Syilx Okanagan Nation.  AS’s doctoral research is situated in the science and practice of integrated knowledge translation. She aims to support spinal cord injury researchers to engage in meaningful research partnerships. |
| Sarah Lawrason | University of British Columbia | Researcher  Trainee | SL is a Postdoctoral Fellow under the supervision of KMG at the University of British Columbia Okanagan. She is also a Researcher with the Public Health Agency of Canada. SL is a white, cis-gender, straight, able-bodied woman, who lives and works on the traditional territory of many nations including the Mississaugas of the Credit, the Anishnabeg, the Chippewa, the Haudenosaunee and the Wendat peoples. Toronto is also covered by Treaty 13 with the Mississaugas of the Credit.  SL’s research focuses on working in partnership with research users to develop, implement, and evaluate resources for individuals with disabilities and chronic conditions. |
| Kathleen Martin Ginis | University of British Columba | Researcher | KMG is a Professor in the Department of Medicine (Division of Physical Medicine and Rehabilitation) and in the School of Health and Exercise Sciences at The University of British Columbia. KMG was the Principal Investigator of CDPP. She identifies as a white, cis-gendered, able-bodied woman of settler ancestry living and working on the traditional and unceded territory of the Syilx Okanagan Nation.  The focus of KMG’s research is on understanding and changing physical activity behaviour. She has a particular interest in physical activity among people living with physical disabilities. She is deeply committed to knowledge translation; specifically, the development and implementation of evidence-based best-practices to improve health and well-being among people with disabilities. |
| Veronica Allan | Sport Information Resource Centre (SIRC) | Researcher | VA is the Research and Innovation Lead Canada’s Sport Information Resource Centre (SIRC), a national non-profit organization that provides research and evaluation services, shares news and information, and mobilizes knowledge for the Canadian sport system. VA uses she/her pronouns and identifies as a white, cis-gendered, able-bodied woman of settler ancestry living and working on the traditional and unceded territory of the Algonquin Anishinaabe Peoples.  Prior to her current role, VA completed her PhD as a trainee in the CDPP network studying meanings of quality participation and effective coaching in disability sport. She also completed a postdoctoral fellowship at York University which included a focus on building capacity for research partnerships and evaluation in national sport organizations. |
| Anita Kothari | Western University | Researcher | AK is a cis-gendered, able-bodied woman of colour who acknowledges the Anishinaabek, Haudenosaunee, Lūnaapéewak and Chonnonton Nations, whose traditional territories are where she lives and works. AK is also a Professor in the School of Health Studies, Faculty of Health Sciences at Western University. AK teaches a graduate level knowledge translation course and conduct research in the area of integrated knowledge translation. These days AK’s research focuses on processes of IKT, ie, how we work together to produce knowledge that is useful, usable and used. Additional information can be found here: <https://www.uwo.ca/fhs/shs/about/faculty/kothari_a.html> |
| Christopher B. McBride | SCI British Columbia  SCI Canada | Leadership role in SCI community organization | CM is the Executive Director of Spinal Cord Injury BC,a charitable non-profit community delivery organization that supports people with spinal cord injuries and their families. CM is a white, cis-gender, straight, able-bodied man of settler ancestry living and working on the traditional territories of the Coast Salish Peoples, in particular, the x məθkwə əm (Musqueam), ʷy̓Skwxwú7mesh (Squamish) and Sə ílwəta /Selilwitulh l̓ʔ(Tsleil-Waututh) First Nations.  He is a research user and community research partner on a broad spectrum of research topics, including in the areas of Behaviour Change and Integrated Knowledge Translation. He partners in research that is relevant and meaningful to the mission of the organization he leads and to the people it serves |
| Heather L. Gainforth | University of British Columbia | Researcher | HG is an Associate Professor at the University of British Columbia and an ICORD Principal Investigator. HG is a white, cis-gender, straight, able-bodied woman of settler ancestry living and working on the traditional and unceded territory of the Syilx Okanagan Nation.  She researches in the areas of Behaviour Change and Integrated Knowledge Translation. Her research aims to combat tokenism in science and foster meaningful engagement between researchers and research users by advancing the science and practice of integrated knowledge translation. |
