# Supplementary material for: Experiences on the implementation and maintenance of the Canadian Disability Participation Project: A mixed-methods study
Source: PLoS One. 2025 Nov 13;20(11):e0334835. doi: 10.1371/journal.pone.0334835 (PMC12614619; doi:10.1371/journal.pone.0334835)
Supplement: S2 Table — (DOCX) [file pone.0334835.s002.docx]

**S2 Table. Our collaborative research activities**

| **Research phase** | **Dates** | **Activity** | **Description** |
| --- | --- | --- | --- |
| Conceptual design | 2018 | Survey development | FH and HG developed the first draft of the survey. KMG, VA, AK provided written feedback on the surveys. CB pilot tested the survey via ‘think-aloud’ process and provided feedback.  FH finalized the survey based on feedback from team members and other people who pilot tested the survey. |
|  | 2018 | Interview guide development | FH drafted the first version of the interview guide. HG and VA provided feedback on the guide. |
|  | 2021 | Interview guide development | FH and SL revised the original developed interview guide. VA, HG and AK provided feedback. The revised interview guide was then pilot tested by VA and finalized after conducting the first interview session.  KMG and CB were not involved in the development of the interview guide as they were invited to take part as a participant in the interviews. |
| Data collection | 2019 – 2021 | Recruitment of participant | FH, KMG and CM assisted with promoting the survey among CDPP members and recruitment of participants for completing the annual survey. |
|  | 2021 - 2022 | Conducting interviews | VA, FH and SL conducted the interviews |
| Data analyses and interpretation | 2022 | Survey data analyses | FH analyzed the survey data and discussed results with VA, AK, KMG and CM. Based on feedback from VA and AK and after a team meeting between with FH, KMG and CM, we decided to focus the manuscript only on implementation-focused survey data. Survey data related to effectiveness and adoptions will be available elsewhere. |
|  |  | Interview data analyses | AS analyzed the interview data and constructed initial themes and sub-themes.  FH, SL, AS and HG met to discuss the themes and findings. Based on the discussion, AS refined the themes. FH, SL, AS and HG met again to further discuss the discussed and refined and renamed the themes during a second meeting. A draft of the refined themes was shared with KMG, CM, VA, and AK, who acted as ‘critical friends’. Based on several rounds of written feedback from co-authors, themes and descriptions were further refined. |
|  | 2024 | Interview data analyses – finalization | FH finalized themes and descriptions after feedback from HG, and co-authors AL, SL, KMG, CM, VA and AK, |
|  | June 2024 | Finalize paper | All co-authors reviewed and approved the paper for submission. |
