# Supplementary material for: Experiences on the implementation and maintenance of the Canadian Disability Participation Project: A mixed-methods study
Source: PLoS One. 2025 Nov 13;20(11):e0334835. doi: 10.1371/journal.pone.0334835 (PMC12614619; doi:10.1371/journal.pone.0334835)
Supplement: S3 Table — (DOCX) [file pone.0334835.s003.docx]

**S4 Table. Disaggregated survey findings on implementation component**

| **CDPP survey items** | **2018** | | | **2019** | | | **2021** | | |
| --- | --- | --- | --- | --- | --- | --- | --- | --- | --- |
|  | Researcher  (n=16) | Trainee  (n=20) | RU  (n=5) | Researcher  (n=13) | Trainee  (n=10) | RU  (n=6) | Researcher  (n=18) | Trainee  (n=9) | RU  (n=6) |
| ***Network functioning*** |  |  |  |  |  |  |  |  |  |
| The **goal** of the CDPP network is clear. | 6.3 ± 1.0 | 6.4 ± 0.8 | 6.6 ± 0.5 | 6.1 ± 1.1 | 6.3 ± 0.8 | 6.3 ± 0.5 | 5.4 ± 1.6 | 5.7 ± 1.4 | 6.0 ± 1.3 |
| The **value** of the CDPP network is clear. | 6.1 ± 1.2 | 6.4 ± 0.8 | 6.2 ± 0.8 | 6.4 ± 0.8 | 6.5 ± 1.3 | 6.5 ± 0.8 | 5.6 ± 1.5 | 5.7 ± 1.4 | 6.0 ± 1.3 |
| It is **easy** to work together with community partners/researchers in the CDPP network. | **4.4 ± 1.4** | **5.5 ± 1.5** | **6.2 ± 0.8** | **5.3 ± 1.5** | **6.1 ± 0.7** | **6.8 ± 0.4** | **5.0 ± 1.5** | **4.8 ± 2.0** | **6.8 ± 0.4** |
| It is **useful** to work together with community partners/researchers in the CDPP network. | 6.4 ± 0.9 | 6.4 ± 1.2 | 6.8 ± 0.4 | 6.5 ± 0.7 | 6.7 ± 0.7 | 6.8 ± 0.4 | 6.3 ± 0.7 | 5.9 ± 2.0 | 6.3 ± 0.8 |
| ***Network satisfaction*** |  |  |  |  |  |  |  |  |  |
| I am satisfied with the way the people and organizations in the CDPP network work together. | 5.1 ± 1.3 | 5.9 ± 1.3 | 5.8 ± 0.8 | 5.8 ± 1.0 | 5.6 ± 1.4 | 6.4 ± 0.9 | 5.3 ± 1.4 | 5.4 ± 1.7 | 6.3 ± 0.8 |
| I am satisfied with **my influence** in the CDPP network. | **5.3 ± 1.3** | **5.0 ± 1.8** | **6.4 ± 1.3** | **5.6 ± 1.4** | **5.1 ± 1.4** | **7.0 ± 0.0** | **4.8 ± 1.5** | **5.0 ± 1.9** | **6.3 ± 1.2** |
| I am satisfied with **my role** in the CDPP network. | 5.9 ± 1.2 | 5.1 ± 1.7 | 6.4 ± 1.3 | **5.8 ± 1.3** | **5.7 ± 1.4** | **6.8 ± 0.4** | **5.0 ± 1.5** | **5.1 ± 1.6** | **6.3 ± 1.0** |
| I am satisfied with the CDPP’s plans for achieving its goals. | 5.9 ± 1.2 | 5.9 ± 1.3 | 6.4 ± 0.5 | 5.7 ± 1.2 | 5.7 ± 1.1 | 6.8 ± 0.5 | 5.4 ± 1.2 | 5.2 ± 1.8 | 6.6 ± 0.5 |
| I am satisfied with the way the CDPP implemented its plans. | 5.7 ± 1.2 | 5.9 ± 1.3 | 6.6 ± 0.5 | 5.7 ± 1.2 | 5.9 ± 1.2 | 6.7 ± 0.5 | 5.6 ± 1.2 | 5.8 ± 1.3 | 6.6 ± 0.5 |

Table 4.1: Members’ views on network functioning and satisfaction (implementation) presented by researchers, trainees and research users.

*Note:* Survey items were rated on a 7-point Likert scale, in which 1 = strongly disagree, 4 = neither disagree nor agree, and 7 = strongly agree. The number of participants who responded ‘unsure’ were excluded from these analyses, as we assumed that these participants were unable to comment on the item (for example due to their limited involvement or role in the network). The percentage of participants who responded ‘unsure’ is listed for each item.

**Response rates for surveys were:**

- 2018: Researchers – 59% (16/27); Trainees – 53% (20/38); Research users – 30% (5/15)
- 2019: Researchers – 48% (13/27); Trainees – 27% (10/37); Research users – 24% (6/25)
- 2021: Researchers – 64% (18/28); Trainees – 36% (9/25); Research users – 54% (6/11)
