# Supplementary material for: Experiences on the implementation and maintenance of the Canadian Disability Participation Project: A mixed-methods study
Source: PLoS One. 2025 Nov 13;20(11):e0334835. doi: 10.1371/journal.pone.0334835 (PMC12614619; doi:10.1371/journal.pone.0334835)

**S3 Text. Interview guide**


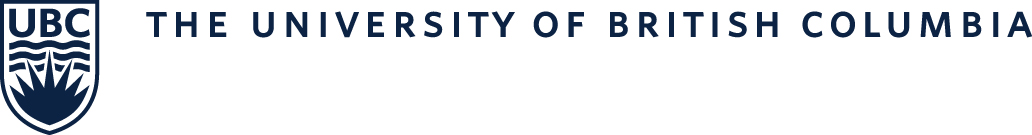


**Evaluating the impact of a network of research partnerships:**

**A longitudinal multiple case study**

**Interview Guide:**

**Introduction**

*Femke:* My name is Femke Hoekstra and I am a postdoctoral research fellow at the University of British Columbia Okanagan.

*Sarah:* My name is Sarah Lawrason and I am a PhD student at the University of British Columbia.

*Veronica:* My name is Veronica Allan and I am a postdoctoral fellow at York University.

*Heather:* My name is Heather Gainforth and I am an associate professor at the University of British Columbia Okanagan.

Thank you for your interest in participating in our study, in which we aim to gain better understanding of how, when, with whom and why research partnerships are successful (or not) in conducting and/or disseminating research together within a large network, such as CDPP. For this project, I am/ we are working under the supervision of Dr. Heather Gainforth and Prof. Dr. Kathleen Martin Ginis in UBC Okanagan’s School of Health and Exercise Sciences.

Before we begin, would you like to change your Zoom name to a nickname or alternative name to have more anonymity while we record?

***Introduction for participants who have previously filled out the annual survey:***

I wanted to check with you if you would like to be reminded of any of the components of the consent form?

**Consent**

Your participation in this study is completely voluntary and you are free to not answer any of the questions. You may withdraw from this study at any time without any consequences.

Before moving forward,

1) Do you have any further questions or would like any additional details of the study?

2) Do you consent to participate knowing you may withdraw at any point with no consequences?

Please confirm that you consent to having this conversation recorded.

**Participant ID Code**

In order to maintain confidentiality, we will be creating a unique ID code for each participant.

1) What are the first three letters of your mother’s maiden name?

2) What are the two digits of your day of birth?

3) What are the last two digits of your telephone number?

**Interview Script**

**Part A.1 – Experiences with CDPP network (Study 1+ 2)**

1. Tell me about your experiences being a part of the CDPP network.
   1. What was your role?
   2. What kind of CDPP projects were you involved in?
2. If any, what are, in your opinion, some of the successes of the CDPP network?
3. If any, what are, in your opinion, some of the challenges of the CDPP network?
   1. How have you learned from these challenges?

**Part A.2 – CDPP network structure over time (Study 1)**

*[The interviewer shows the results of the network analysis over time to the participants. The interviewer shows the network figures of the CDPP and explains the network figures to the participants].*

*See visuals in powerpoint slide (or below)*

*The network figures that we are showing you are visualizations of the entire CDPP network. Each icon represents a person, whether it be a researcher, community partner, or trainee. The icons are different colours/shapes to represent each person’s role. Orange squares are researchers, green circles are community partners, yellow triangles are community partners and researchers, and blue triangles are trainees. Each line represents a connection between two people – or that these two people directly worked on a project together.*

*The first figure shows connections of projects conducted between the years 2014-2016. The second figure shows connections of projects between the years 2017-2021. The third figure shows overall connections between the years 2014 – 2021. These projects are either published academic articles or formal KT products listed on the CDPP Team CVs.*

*We created this figure using the following procedures:*

- *We identified the lead academic researcher (i.e., assistant or associate or full professor) for each CDPP publication/KT product via the first, last or second listed author on the reference.*
- *We created connections between the lead researcher and all their co-authors and collaborators for each listed year. The lead researcher reviewed and added this information via their personalized CDPP KT Activities Report.*

*As a result, you will only see lead researchers (and not trainees or community partners) in the core of the network. The visuals may therefore not reflect the actual collaborations and interactions that have been taken place within the CDPP network. While we acknowledge the limitations of our procedures, we would like to use these visuals to guide the next part of this interview. [The interviewer mentions who the interviewee is in the network].*

1. Please take a moment to look at the two different network figures What are your impressions of the network figure?
2. What changes in the CDPP network structure over time do you see?
3. What do you think are possible explanations for these changes?
   1. Can you provide an example?

**Part A.3 – Your role in CDPP network (Study 1)**

*[The interviewer illustrates participant’s role in the CDPP network based on the network figures – from past to present]*

1. How well do these network figures resonate with your experience in the network? Why (not)?
   1. Possible probe: Would you have described the network differently before you saw these figures?
2. Based on your own experiences, how do you feel about your current role in the CDPP network?
   1. How has your role changed over time in the CDPP? How do you feel about these changes (or no changes)?
   2. What changes to the network, if any, do you think may be helpful to improve KT capacity??

**Part A.4 – closing questions (Study 2)**

1. What lessons did you learn from being part of the CDPP network?
2. In your opinion, how should CDPP network function after 5 years (2026)?

**PART B.1 – Partnership experiences**

In the second part of this interview, we would like to hear more about your research partnership experiences within the CDPP partnership.

1. Can you tell me more about your experiences with conducting and/or disseminating CDPP research [insert project] in partnership with research users?
2. If you think about this CDPP partnership project, what lessons did you learn from this CDPP partnership project?
   1. What worked for you in this partnership?
   2. What did not work for you in this partnership?
   3. What kinds of supports do you think would help a partnership that is aiming to translate research findings to non-academic audiences?

**Part B.2: Closing questions**

1. In your opinion, what is a successful partnership?
2. If you had a colleague ask you about doing research in partnership with research users, what types of recommendations would you share?
3. ***If time (not necessary):***
   1. What principles or values are important to you when conducting and disseminating research in partnership?
      1. Probe: If your partner was answering this question, what would they say?
   2. What factors support or hinder the engagement of community partners throughout the research process?
      1. Probe: Did your partner(s) use any strategies that helped to engage users in your research project?
4. Do you have anything you would like to add or any questions that I can address?


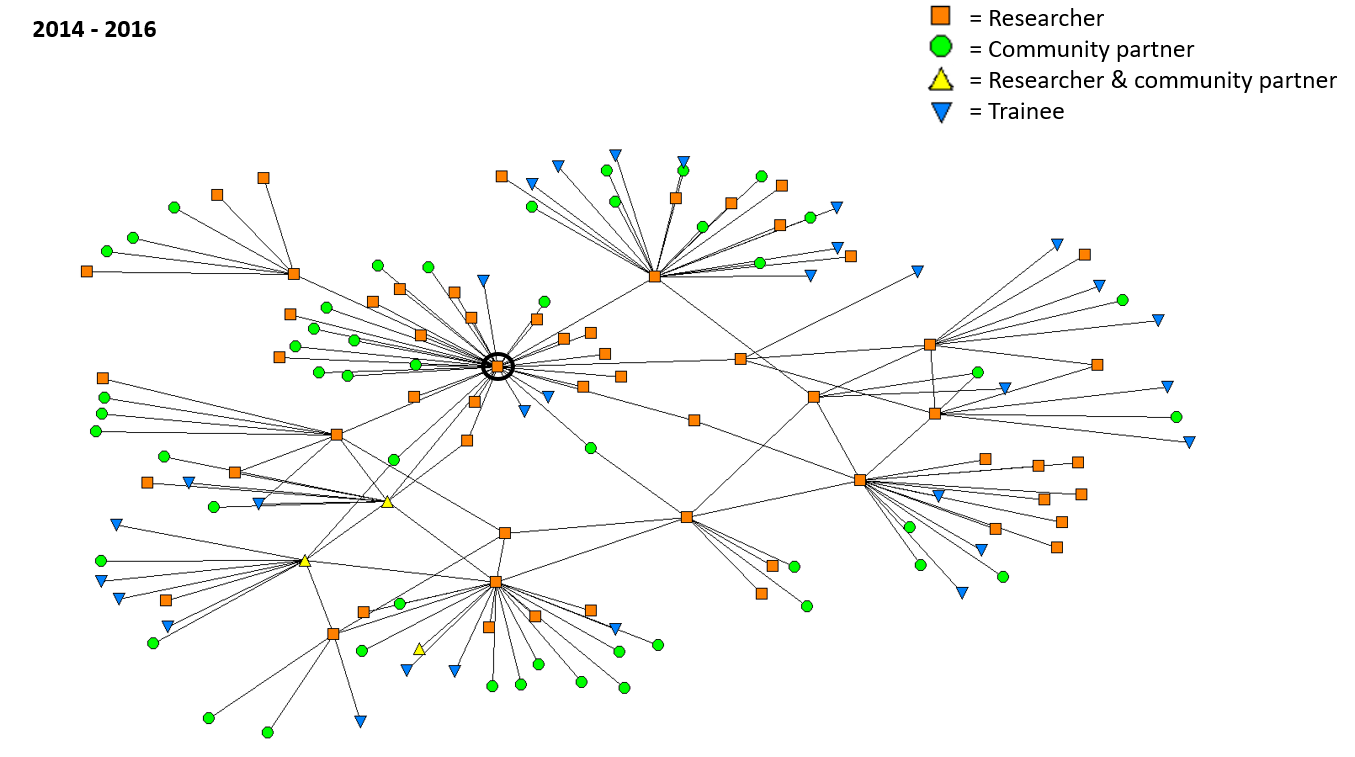


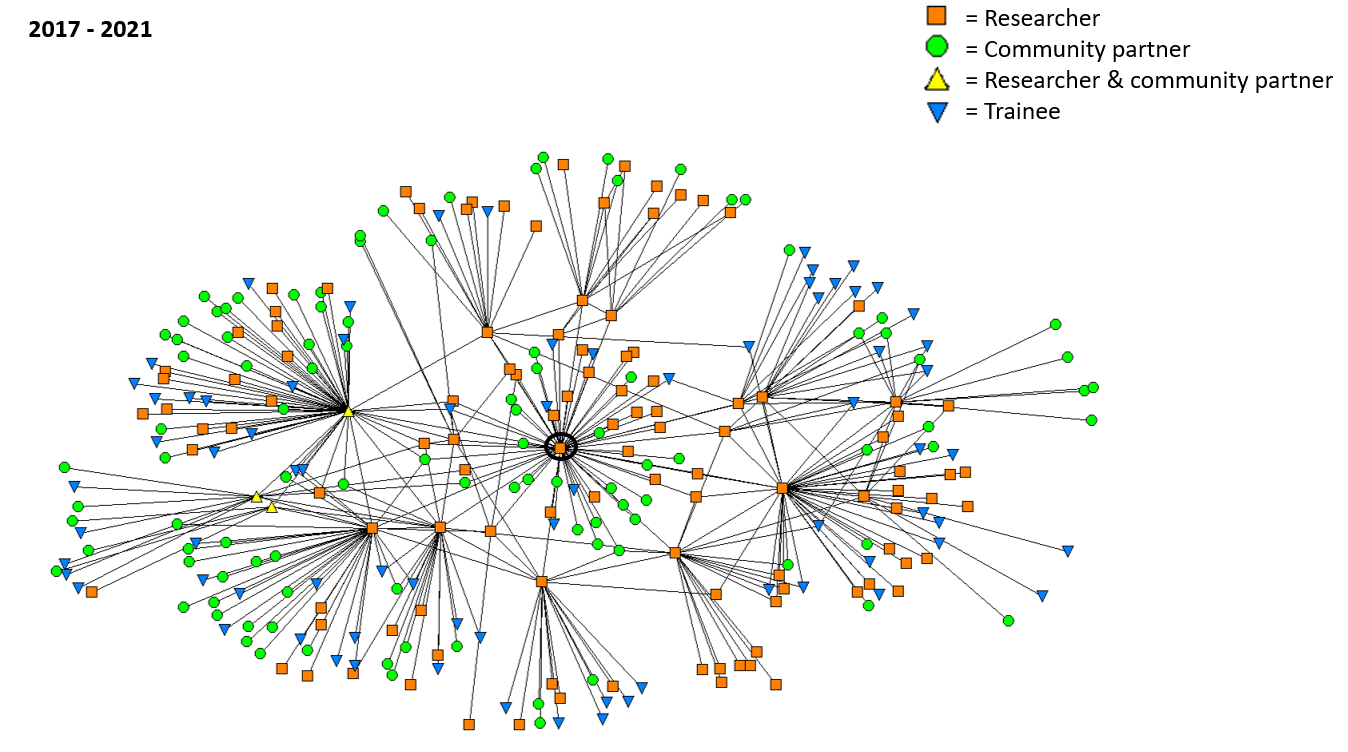


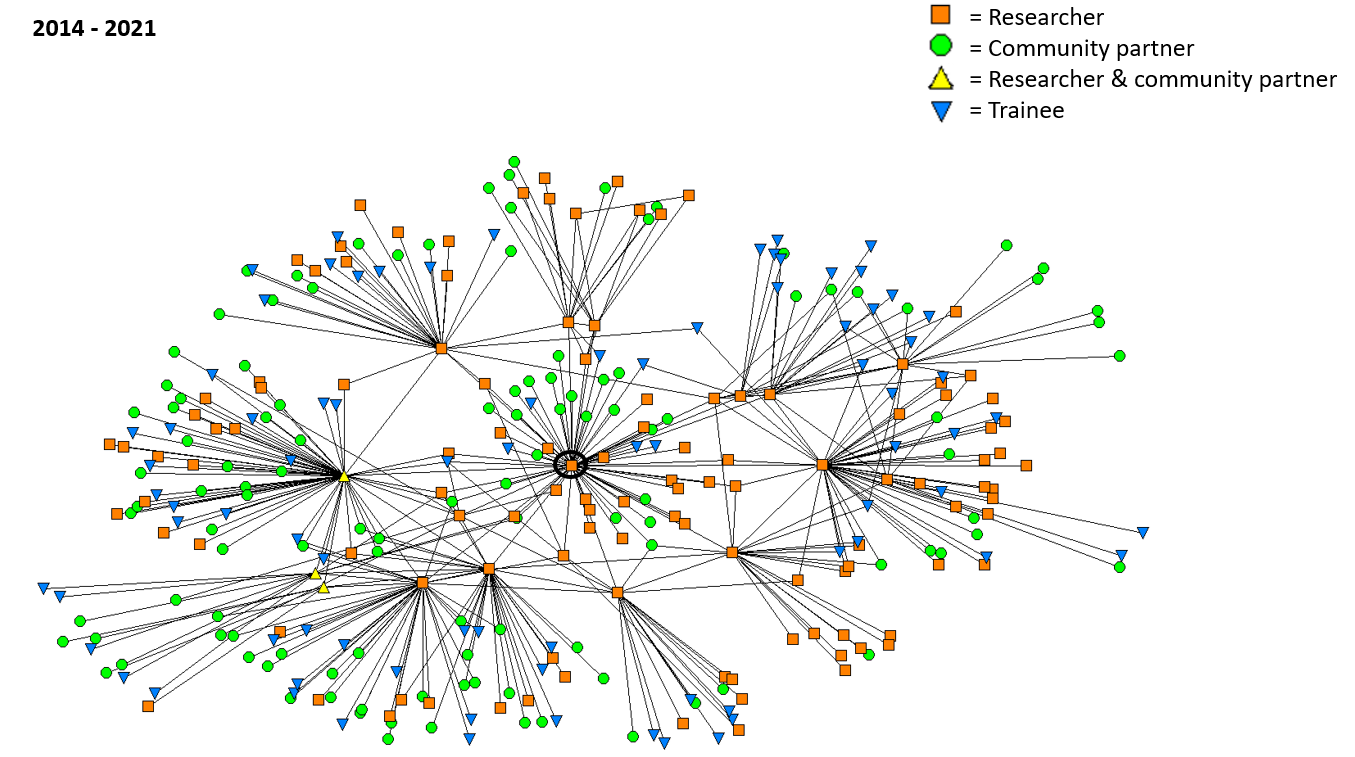

Supplement: S1 Text — (DOCX) [file pone.0334835.s005.docx]
