# Supplementary material for: Experiences on the implementation and maintenance of the Canadian Disability Participation Project: A mixed-methods study
Source: PLoS One. 2025 Nov 13;20(11):e0334835. doi: 10.1371/journal.pone.0334835 (PMC12614619; doi:10.1371/journal.pone.0334835)
Supplement: Attachment — Submitted filename: 250730_CDPP-Responses-to-Reviewers.docx [file pone.0334835.s007.docx]

Journal Requirements:

When submitting your revision, we need you to address these additional requirements.

Thank you for the valuable comments on our manuscript. We have carefully addressed each of the reviewer’s comments, and our responses are provided in blue text. If only parts of the paragraph has been changed, we have highlighted these changes in yellow. To support our responses, we have included the relevant revised paragraphs from the manuscript within this response letter. All changes to the manuscript are reflected using the Track Changes feature in Microsoft Word.

1. Please ensure that your manuscript meets PLOS ONE's style requirements, including those for file naming. The PLOS ONE style templates can be found at

<https://journals.plos.org/plosone/s/file?id=wjVg/PLOSOne_formatting_sample_main_body.pdf> and

[https://journals.plos.org/plosone/s/file?id=ba62/PLOSOne_formatting_sample_title_authors_affiliations.pdf](https://track.editorialmanager.com/CL0/https:%2F%2Fjournals.plos.org%2Fplosone%2Fs%2Ffile%3Fid=ba62%2FPLOSOne_formatting_sample_title_authors_affiliations.pdf/1/010f01978244eb8b-ff35a352-807b-4b84-8e2e-788e8f31a3ca-000000/8HB_gmBa1SiIPcUOoJJUR6i897XgG0qIiw1iDc9XhyA=216)

We have made the necessary changes to ensure the manuscript meets the journal’s formatting requirements. Please note that these formatting updates are not tracked using the “Track Changes” feature.

The changes include:

1. The title page was updated to reflect the journal’s formatting guidelines, including author contributions.
2. The abstract heading size was adjusted, subheadings were removed, and the abstract was reformatted into a single paragraph, following the example provided in the main body formatting link.
3. Headings in the main body of the manuscript were reformatted (e.g., font size and heading levels) according to the provided guidelines.
4. Square brackets were used in place of round brackets for in-text citations throughout the manuscript.

2. Thank you for stating the following financial disclosure:

This study was supported by a partnership grant from the Social Sciences and Humanities Research Council of Canada (grant no. 895-2013-1021) for the Canadian Disability Participation Project ([www.cdpp.ca](http://track.editorialmanager.com/CL0/http:%2F%2Fwww.cdpp.ca/1/010f01978244eb8b-ff35a352-807b-4b84-8e2e-788e8f31a3ca-000000/ISmgtKuipcO0P1vlFQQif7s2Xel6cRpfznSJvswUfr0=216)).

Please state what role the funders took in the study. If the funders had no role, please state: "The funders had no role in study design, data collection and analysis, decision to publish, or preparation of the manuscript."
If this statement is not correct you must amend it as needed.
Please include this amended Role of Funder statement in your cover letter; we will change the online submission form on your behalf.

This is correct. We added the statement that the funder had no role in the study design, data collection and analysis, decision to publish, or preparation of the manuscript to the cover letter.

3. When completing the data availability statement of the submission form, you indicated that you will make your data available on acceptance. We strongly recommend all authors decide on a data sharing plan before acceptance, as the process can be lengthy and hold up publication timelines. Please note that, though access restrictions are acceptable now, your entire data will need to be made freely accessible if your manuscript is accepted for publication. This policy applies to all data except where public deposition would breach compliance with the protocol approved by your research ethics board. If you are unable to adhere to our open data policy, please kindly revise your statement to explain your reasoning and we will seek the editor's input on an exemption. Please be assured that, once you have provided your new statement, the assessment of your exemption will not hold up the peer review process.

The survey data as well as other supporting documents (survey questions and interview guide) is now publicly available via Open Science Framework: <https://osf.io/rwf2u/>

The direct link to the de-identified survey data is: <https://osf.io/rwf2u/files/osfstorage>

However, the interview transcripts cannot be shared due to ethical considerations, as they may contain identifiable information about participants. We can provide additional quotes from the interview data upon reasonable request.

4. We note that you have indicated that there are restrictions to data sharing for this study. For studies involving human research participant data or other sensitive data, we encourage authors to share de-identified or anonymized data. However, when data cannot be publicly shared for ethical reasons, we allow authors to make their data sets available upon request. For information on unacceptable data access restrictions, please see <http://journals.plos.org/plosone/s/data-availability#loc-unacceptable-data-access-restrictions>.

As indicated in the previous comment, the survey data is publicly available via Open Science Framework. However, the interview transcripts cannot be shared due to ethical considerations, as they may contain identifiable information about participants. We can provide additional quotes from the interview data upon reasonable request.

Before we proceed with your manuscript, please address the following prompts:

a) If there are ethical or legal restrictions on sharing a de-identified data set, please explain them in detail (e.g., data contain potentially identifying or sensitive patient information, data are owned by a third-party organization, etc.) and who has imposed them (e.g., a Research Ethics Committee or Institutional Review Board, etc.). Please also provide contact information for a data access committee, ethics committee, or other institutional body to which data requests may be sent.

There are ethical restrictions on sharing the interview transcripts, as they may contain identifiable information. Given the nature of the research topic, where participants discuss their own research projects and partnerships, it may be possible to identify participants’ identities. These restrictions are in accordance with the regulations of the Behavioural Research Ethics Board of the University of British Columbia Okanagan that reviewed and approved this study.

b) If there are no restrictions, please upload the minimal anonymized data set necessary to replicate your study findings to a stable, public repository and provide us with the relevant URLs, DOIs, or accession numbers. Please see <http://www.bmj.com/content/340/bmj.c181.long> for guidelines on how to de-identify and prepare clinical data for publication. For a list of recommended repositories, please see <https://journals.plos.org/plosone/s/recommended-repositories>. You also have the option of uploading the data as Supporting Information files, but we would recommend depositing data directly to a data repository if possible.

Please update your Data Availability statement in the submission form accordingly.

The survey data is available on Open Science Framework via the following link: <https://osf.io/rwf2u/files/osfstorage>

Open Science Framework is listed as one the acceptable cross-disciplinary repositories.

5. Thank you for uploading your study's underlying data set. Unfortunately, the repository you have noted in your Data Availability statement does not qualify as an acceptable data repository according to PLOS's standards.

At this time, please upload the minimal data set necessary to replicate your study's findings to a stable, public repository (such as figshare or Dryad) and provide us with the relevant URLs, DOIs, or accession numbers that may be used to access these data. For a list of recommended repositories and additional information on PLOS standards for data deposition, please see [https://journals.plos.org/plosone/s/recommended-repositories](https://track.editorialmanager.com/CL0/https:%2F%2Fjournals.plos.org%2Fplosone%2Fs%2Frecommended-repositories/1/010f01978244eb8b-ff35a352-807b-4b84-8e2e-788e8f31a3ca-000000/wTLpTKapuCxUQuUZE4c5vH8eEZLt7d38Q7O1wV5e4uc=216).

The survey data is publicly available via Open Science Framework, which is listed as one of the recommended repositories.

6. [Please remove all personal information, ensure that the data shared are in accordance with participant consent, and re-upload a fully anonymized data set.

Note: spreadsheet columns with personal information must be removed and not hidden as all hidden columns will appear in the published file.

Additional guidance on preparing raw data for publication can be found in our Data Policy (https://journals.plos.org/plosone/s/data-availability#loc-human-research-participant-data-and-other-sensitive-data) and in the following article: http://www.bmj.com/content/340/bmj.c181.long.](http://track.editorialmanager.com/CL0/http:%2F%2Fwww.bmj.com%2Fcontent%2F340%2Fbmj.c181.long./1/010f01978244eb8b-ff35a352-807b-4b84-8e2e-788e8f31a3ca-000000/lcPU-JrbKYJHpsZgdGDd7VFyx1UPEtAurDUhL4cJ9fI=216)

The survey data with personal information removed is available on Open Science Framework via the following link: <https://osf.io/rwf2u/files/osfstorage>

7. [Please include captions for your Supporting Information files at the end of your manuscript, and update any in-text citations to match accordingly. Please see our Supporting Information guidelines for more information: http://journals.plos.org/plosone/s/supporting-information.](https://ubcca-my.sharepoint.com/personal/adrienne_sinden_ubc_ca/Documents/-Femke%20Hoekstra%202024/CDPP1.0%202025%20paper/Please%20include%20captions%20for%20your%20Supporting%20Information%20files%20at%20the%20end%20of%20your%20manuscript,%20and%20update%20any%20in-text%20citations%20to%20match%20accordingly.%20Please%20see%20our%20Supporting%20Information%20guidelines%20for%20more%20information:%20http:/journals.plos.org/plosone/s/supporting-information.)

As per the guidelines in the link provided above, a supporting information heading was created at the end of the last page of the manuscript and all supporting information is listed as per the guidelines below the heading. Separate supporting information files were made and named according to the guidelines.

[Note: HTML markup is below. Please do not edit.]

Reviewers' comments:

Reviewer's Responses to Questions

**Comments to the Author**

1. Is the manuscript technically sound, and do the data support the conclusions?

The manuscript must describe a technically sound piece of scientific research with data that supports the conclusions. Experiments must have been conducted rigorously, with appropriate controls, replication, and sample sizes. The conclusions must be drawn appropriately based on the data presented.

Reviewer #1: Yes

Reviewer #2: Yes

2. Has the statistical analysis been performed appropriately and rigorously?

Reviewer #1: Yes

Reviewer #2: Yes

3. Have the authors made all data underlying the findings in their manuscript fully available?

The [PLOS Data policy](http://track.editorialmanager.com/CL0/http:%2F%2Fwww.plosone.org%2Fstatic%2Fpolicies.action%23sharing/1/010f01978244eb8b-ff35a352-807b-4b84-8e2e-788e8f31a3ca-000000/qa8ik2ZGEPF7hus9pFW9fvtnHX_FUh_328cgTJIUXnE=216) requires authors to make all data underlying the findings described in their manuscript fully available without restriction, with rare exception (please refer to the Data Availability Statement in the manuscript PDF file). The data should be provided as part of the manuscript or its supporting information, or deposited to a public repository. For example, in addition to summary statistics, the data points behind means, medians and variance measures should be available. If there are restrictions on publicly sharing data—e.g. participant privacy or use of data from a third party—those must be specified.

Reviewer #1: Yes

Reviewer #2: Yes

4. Is the manuscript presented in an intelligible fashion and written in standard English?

PLOS ONE does not copyedit accepted manuscripts, so the language in submitted articles must be clear, correct, and unambiguous. Any typographical or grammatical errors should be corrected at revision, so please note any specific errors here.

Reviewer #1: Yes

Reviewer #2: Yes

5. Review Comments to the Author

Please use the space provided to explain your answers to the questions above. You may also include additional comments for the author, including concerns about dual publication, research ethics, or publication ethics. (Please upload your review as an attachment if it exceeds 20,000 characters)

**Reviewer #1:** Thank you for the opportunity to review this manuscript, which explores the implementation and sustainability of the CDPP network through a mixed-methods design. The focus on partnership-based research networks in the field of disability studies is timely and relevant, particularly as collaborative and interdisciplinary approaches continue to grow.
Below are detailed comments aimed at enhancing the clarity, coherence, and overall contribution of the manuscript:

a. The second objective was meant to explore both network-level and partnership-level experiences. However, because of a change in methods, both levels were covered in one interview session. While this change is understandable, the paper does not fully explain how it may have affected the results. This shift might have made it harder to clearly understand partnership-level sustainability, which is an important part of the study. It would be helpful if the authors discussed how this change may have shaped the detail and focus of the findings.

The reviewer is correct that the shift in methodology, focusing on both the network-level and project-level, had implications for our ability to gather in-depth information about individual project-level partnerships and their sustainability. While participants did share their experiences of working together within specific CDPP partnership projects, we did not collect longitudinal data at the project level, nor were we able to interview all or multiple individuals involved in each specific partnership. This limited our ability to gain a comprehensive understanding of individual partnerships and prevented us from conducting a true multiple case study as originally intended (Hoekstra et al. 2018).

However, we did collect rich, longitudinal data on the CDPP partnership at the network level, which is described in detail in the manuscript. Through the continuation of the CDPP 2.0 network, we further plan to actively collect additional data on both the sustainability of the network and individual project-level partnerships.

To address this limitation, we have added the following paragraph to the discussion section of the paper (page 32).

*Another limitation is the limited depth of data collected at the individual project level, as interviews focused on both network-level and project-level experiences and were conducted at a single time point. While participants shared valuable insights into their CDPP projects and partnerships, this design limited our ability to conduct the planned longitudinal multiple case analysis. Future follow-up interviews could be conducted within the context of CDPP 2.0 to enhance our understanding of how project-level partnerships are sustained over time.*

b. The paper introduces common challenges to partnerships, such as time pressures, communication issues, and funding problems. However, these do not appear in the findings. The authors should clarify whether participants did not mention these issues, or if they were raised but left out of the analysis, and explain why.

While participants did mention various challenges, we decided to phrase the results section on the positive aspects, specifically, what is needed for success and what contributed to the effective implementation of the CDPP partnership, instead of focusing on challenges explicitly.

Please note that throughout the narrative examples of challenges are discussed in the context of what support was needed. For example:

a. **Challenge of working with different teams (page 18)**
   *One researcher mentioned that they underestimated how difficult it would be to bring together researchers from different areas:*

*“I think I underestimated how difficult it would be to bring those three streams together…the challenges were that I was working with three different streams…we're working in three very different scientific contexts…*

b. **Challenge of to foster cohesion (page 19)**

*Although participants perceived the growth of the CDPP as impressive and helpful for advancing collaborations and partnerships, participants mentioned it was difficult to foster cohesion among a network of this size. One researcher explained that the sheer size of the network makes it difficult to unite the CDPP as one group:*

*“The size of it is a strength, but I’d say it’s also a limitation…”*

c. **Challenge of communication (page 19)**

*A community research user explained that communication through a large entity like the CDPP is challenging.*

Given that many existing studies already highlight barriers and challenges to research partnership approaches, our contribution to the literature is the identification of key elements that support the sustainable implementation of a large-scale partnership network involving both research and community partners. Rather than emphasizing challenges explicitly, we chose to focus on successful elements and the types of support required for both implementation and long-term sustainability of large network of partnerships, like the CDPP.

c. The idea that strong networks can help research influence policy and practice is convincing, but the paper does not include any examples. If there are examples of research from the network being used in practice, it would strengthen the argument to include them. If there were no such cases, the authors might consider adjusting the conclusion to reflect that. Were there any cases where research led to changes in clinical work, community programs, or policies?

Thank you for pointing this out. There are several examples of how CDPP research findings have been successfully translated into policy and practice, also illustrated by the publication of over 100 knowledge mobilization resources and products available on the CDPP website (www.cdpp.ca). While we do not have systematic data on the extent to which these KM resources are being used in practice or policy, we do have illustrative examples of their impact across various domains. For example, the Blueprint for Building Quality Participation in Sport for Children, Youth, and Adults with a Disability has been widely used by organizations such as the Canadian Paralympic Committee (CPC) to inform inclusive sport programming. CDPP research has also contributed to increased attention on creating effective, equitable, and inclusive workplaces for all individuals, including those with disabilities, by providing employers and organizations with practical resources to support inclusive workplaces.

We acknowledge that we have not explicitly studied whether the partnership itself directly contributed to improved translation of research into practice or policy, as we did not include a comparison group. Therefore, in the concluding statement of the paper, we intentionally use the phrase “may improve the translation of research” to reflect this nuance. The statement reads as follows: “*We describe lessons learned on how networks of researchers and research users may help to build capacity for conducting and disseminating research in partnership, and subsequently may improve the translation of research to practice and policy*.”

To address this comment, we have added a paragraph in the discussion section highlighting the knowledge mobilization resources and examples of how CDPP research has been used in practice and policy. The paragraph reads as follows (page 29/30):

*To illustrate the impact of the CDPP network, over 100 knowledge mobilization resources and products have been published and are available on the CDPP website (www.cdpp.ca), spanning the domains of employment, mobility, and expertise and sport. These resources include blueprints, toolkits, and guides that provide accessible, evidence-informed information for a broad range of audiences. For example, the Blueprint for Building Quality Participation in Sport for Children, Youth, and Adults with a Disability has been widely used by organizations such as the Canadian Paralympic Committee (CPC) to inform inclusive sport programming. CDPP research has also contributed to increased attention on creating effective, equitable, and inclusive workplaces for all individuals, including those with disabilities, by providing employers and organizations with resources to promote inclusive workplaces. The value and continued relevance of CDPP’s research in exercise and sport is further demonstrated by the establishment of the CDPP 2.0 network, which includes 29 community partners who continue to benefit from and contribute to this work.*

d. The paper could benefit from more information on how aspects of the network such as leadership, structure, or ways of engaging people changed over time. This would help readers better understand how the network developed.

The reviewer is correct that the structure, leadership, and engagement of the CDPP may have changed over time. Although survey data were collected at multiple timepoints, we did not track individual roles or leadership positions longitudinally. To support interpretation, contextual information on CDPP network has been provided throughout the manuscript.

For example, in the methods section titled 'The CDPP Network,' (page 8-9) we describe the network’s structure and the leadership roles across the three teams. Despite the network’s growth, this structure has remained consistent throughout the study period. We believe the descriptions provide sufficient context to understand potential variation in network over time.

e. It would also be helpful to explain how the findings were shared with, or used by, network members or community partners.

The survey findings were shared through an infographic and a newsletter. The interviews were conducted at the end of the project period, and we are currently awaiting the publication of the paper before engaging in broader knowledge translation activities. However, the insights gained have already informed the direction of CDPP 2.0 (www.cdpp2.ca). For example, CDPP 2.0 now focuses exclusively on sport and exercise, which was a strategic decision made to address the complexities of collaborating across diverse teams and the varying stages of development (as discussed in Lesson Learned 2 in the discussion section).

Furthermore, our findings highlight the need to build capacity for principle-based partnerships. As a result, training and mentoring have become key components of the CDPP 2.0 network. The third lesson learned emphasizes the importance of shared principles or values to guide partnerships. In CDPP 2.0, the Integrated Knowledge Translation (IKT) Guiding Principles for conducting and disseminating spinal cord injury in partnership are being used to support and guide future partnerships within CDPP 2.0 network. These highlights are described in the “Lessons Learned” section of the discussions as follows:

*1)	Foster transformational leadership behaviours to build capacity for principle-based research partnerships among researchers, trainees and research users. This includes mentoring and training early-career researchers, trainees and research users in becoming transformational leaders in research partnerships. Mentoring and training the future generation is needed to successfully sustain the network and its partnerships over time, and has become a key component of the CDPP 2.0 network.*

*2)	When establishing a multidisciplinary network, consider the stage of partnership development, the feasibility of doing multidisciplinary research, and the potential growth of the network over time. For example, the CDPP decided to move forward as three separate networks. Continuing with networks within the same research domain may help members to feel connected to both the network and their partnerships.*

*3)	Formulate shared principles or values to guide collaborations and partnerships within the network. For example, the IKT Guiding Principles have been developed to support conducting and disseminating spinal cord injury research in partnerships [31]. These principles will also be used to guide and evaluate future partnerships in the CDPP 2.0 network.*

f. They say themes were constructed in discussion with co-authors and critical friends but how were the themes derived?

The first co-author (AS) constructed initial themes. This is also described in data analysis section of the methods section. We have revised the first sentence of the paragraph describing the analyses process of the interviews and this paragraph reads as follows (page 11/12):

*First, co-author (AS) became familiar with the interview data, reviewed the transcripts to identify codes and then constructed initial themes. The coding and construction of initial themes were done by co-author (AS) who did not conduct any of the interviews to minimize the risk that experiences from certain participants would get more attention as interview sessions were conducted by different team members. Second, four co-authors (FH, SL, AS, HG) met to discuss and clarify those initial themes over a 1-hour in-person meeting. After the meeting, AS then further refined, defined, and named a next iteration of themes. The same four co-authors met again for another 1-hour meeting to discuss the refined themes. A draft of refined themes and sample quotes were shared with other co-authors (KMG, CBM, AK, VA) who acted as “critical friends” (i.e., a trusted person who asks challenging questions, another lens, and offers critiques) [35]. Based on their feedback, themes and descriptions were further refined and incorporated into the manuscript. Lastly, the first author (FH) finalized theme names and descriptions based on multiple rounds of feedback from all co-authors.*

g. Please clarify how consistency and credibility were ensured during the early coding stages.

Thank you for this comment. In the section on methodological rigour (page 12 -13), we describe several strategies that we used to enhance the quality and credibility of our study. As noted, we drew on theory and previous literature to interpret and reflect on our findings, which contributed to the credibility and meaningful coherence of our analysis. We also meaningfully engaged research users (e.g., community partners and network leaders) throughout the project, including in the analysis process. This engagement not only enhanced the relevance and usefulness of the findings but also strengthened the credibility of the findings. Furthermore, critical friends (SL, FH, HG) were involved early and throughout the coding and analysis process, and worked closely with the lead coder (AS) to reflect on and challenge interpretations, which further contributed to the credibility of the findings.

Regarding the reviewer’s mention of “consistency,” we note that this is not one of the eight criteria for excellent qualitative research outlined by Tracy et al. (2010). It is unclear how the reviewer is defining consistency in this context.

We conducted a reflexive thematic analysis, which emphasizes researcher reflexivity over concepts such as inter-rater reliability or coding consistency. In this approach, different researchers may interpret and analyze the data in different, yet equally valid, ways. Unlike more deductive approaches, reflexive thematic analysis does not aim for coding consistency across coders. Instead, we prioritized reflexive engagement. As noted, critical friends were involved throughout the process to support reflexivity and enhance the quality and credibility of the analysis.

Our approach aligns with a pragmatic approach and demonstrates methodological coherence, which is also an important criterion for quality in qualitative research, which could also be what the reviewer is referring to when mentioning “consistency.”

Reviewer #2: Review of Experiences on the implementation and maintenance of the Canadian Disability Participation Project: a mixed-methods study

The authors present original research that is a component part of an evaluation of the Canadian Disability Participation Project networking using the RE-AIM framework. According to the authors, “More specifically, this study aimed to:
1) describe the implementation of the CDPP network over time;
2) explore members’ experiences and reflections on the implementation and maintenance of the CDPP network and its partnerships.”

My assessment of this mixed methods research is that the design and methods are generally sound but require additional attention in order to ensure coherence throughout the manuscript and generate conclusions that best reflect the data that were generated.

Below is a list of issues that I identified through my review:

1. Clarity in meaning about Implementation

On page 6, the authors further detail the meanings of the two RE-AIM components that were studied, stating that “Implementation focused on CDPP members’ views on how the network functioned over time and their experiences of being part of the network and its partnerships (lines 107-8).” In their description of the data collection (survey), the authors initially repeat this focus on “views on how the network functioned” and “experiences of being part of” the network. In the lines that follow, the first concept of interest remains consistent while the second becomes “network satisfaction” (p. 10, line 199). This change is not explained, leaving uncertainty about the authors’ focus. I recommend that the authors clarify the second part of the implementation component, either by using consistent descriptions throughout the manuscript or by specifying how experiences and satisfaction are related to one another as part of implementation.

Thank you for highlighting the inconsistency in our manuscript. We agree with the reviewer that satisfaction and experience are distinct constructs. To clarify:

- Network satisfaction was assessed through the annual surveys and is reported as part of the implementation component, aligning with the first aim of the study
- Participants’ experiences of being part of the network and its partnerships were explored through the interviews, aligning with the second aim of the study.

To address this comment and enhance clarity and consistency, we have explicitly referred to network satisfaction as a specific aspect of implementation when discussing survey data, and we have ensured consistent terminology throughout the manuscript.

Changes to the manuscript include

*Implementation focused on CDPP members’ views on how the network functioned, their satisfaction over time and their experiences of being part of the network and its partnerships. (page 6)*

*CDPP members were invited to complete an annual survey about their views on how the network functioned (i.e., implementation) and their satisfaction levels of being part of the CDPP network. (page 10)*

2. Clarity about network membership

It is unclear to me who was considered to be a member of the network and how big the network was. This uncertainty is particularly relevant for research users who are presented as both “CDPP members” (p. 9, line 179) and researchers’ partners (p. 9, line 184).

Clarity about membership is particularly relevant to determine the response rate, an issue that seems to be of concern to the authors given the limitations identified with respect to the small numbers of survey responses from research users and the possibility of a response bias.

CDPP members included researchers, trainees, and research users affiliated with the network. Researchers were defined as co-investigators or collaborators on the original CDPP grant, along with their trainees working on CDPP-related projects. The survey response rates were as follows:

- 2018: Researchers – 59% (16/27); Trainees – 53% (20/38); Research users – 30% (5/15)
- 2019: Researchers – 48% (13/27); Trainees – 27% (10/37); Research users – 24% (6/25)
- 2021: Researchers – 64% (18/28); Trainees – 36% (9/25); Research users – 54% (6/11)

To address the reviewer’s comment, we have added these response rates below Table S4 (Supplementary File 4), which presents the disaggregated survey findings.

The reviewer is also correct in indicating the potential for response bias, as both researchers and research users may have had financial or professional benefits to their involvement in CDPP. This could have influenced them to respond in a socially desirable manner. To mitigate this risk, we ensured that all survey responses were collected anonymously, and we clearly communicated to participants that their responses would remain confidential and would not affect their access to CDPP services or opportunities.

To reflect this, we have added the following sentence to the Limitations section of the manuscript:

*There is a potential for response bias in the survey data, as CDPP members may have felt inclined to provide socially desirable responses due to their professional or financial benefits to the CDPP network. However, this risk was mitigated by collecting anonymous data and emphasizing that participation in the survey would not impact their involvement in CDPP activities or access to services.*

3. From my read of the authors’ materials, the most promising description of the network make-up and size is the figures representing the CDPP network 2014-2021 that are included in the interview guide. Are these accurate portrayals of the numbers and distribution of the people involved? How do the authors attend to a potentially blurred boundary between a research user being a “community partner” or a “network member”?

The network visuals offer a clear representation of the overall structure of the CDPP network and were used as a tool to guide the interview discussions. As outlined in Supplementary File 3, the network figures were generated using the following process:

*The network figures are visualizations of the entire CDPP network. Each icon represents a person, whether it be a researcher, community partner, or trainee. The icons are different colours/shapes to represent each person’s role. Orange squares are researchers, green circles are community partners, yellow triangles are community partners and researchers, and blue triangles are trainees. Each line represents a connection between two people – or that these two people directly worked on a project together.*

*The first figure shows connections of projects conducted between the years 2014-2016. The second figure shows connections of projects between the years 2017-2021. The third figure shows overall connections between the years 2014 – 2021. These projects are either published academic articles or formal KT products listed on the CDPP Team CVs.*

*We created this figure using the following procedures:*

- *We identified the lead academic researcher (i.e., assistant or associate or full professor) for each CDPP publication/KT product via the first, last or second listed author on the reference.*
- *We created connections between the lead researcher and all their co-authors and collaborators for each listed year. The lead researcher reviewed and added this information via their personalized CDPP KT Activities Report.*

*As a result, you will only see lead researchers (and not trainees or community partners) in the core of the network. The visuals may therefore not reflect the actual collaborations and interactions that have been taken place within the CDPP network. While we acknowledge the limitations of our procedures, we would like to use these visuals to guide the next part of this interview.*

As such, the network figures may not reflect the actual collaborations or interactions that took place within the CDPP network.

To address the blurred boundary between researchers and research users, we created a distinct category for individuals who held dual roles. These individuals were involved both as researchers and as research users within the CDPP and were categorized accordingly. In the network figures, they are marked in yellow to accurately reflect their dual contributions and positions within the network.

I also note that in the text, the authors state, “S3 Text provides additional details on how the CDPP operated, including a visual of the governance structure” (p. 9 lines 174-6). I believe that this is an error as S3 contains the interview guide and I was not able to find the governance structure amid the appendices.

Thank you for pointing this out. We have removed the sentence in question, as it referred to an earlier version of the manuscript. The description of the CDPP has been integrated into the main text, and the governance structure is described within the narrative on the CDPP network on pages 8–9.

**4. Clarity in the Maintenance (Capacity building) theme**

The authors present one theme, “Build capacity to sustain,” as a finding from the Maintenance component of the study. Although not explicitly linked, the theme seemed to be related to and supportive of the first theme, “Strong leadership.”

The reviewer is correct that the findings of the themes on implementation and maintenance relate to each other. In our study, we asked participants specific questions about both their experiences during the implementation phase and their perceptions of sustainability over time. As a result, the first theme focuses on experiences and reflections of past experiences during implementation, while the maintenance theme captures participants’ views on how the network could be sustained in the future.

It is possible that participants may have drawn on their past experiences when discussing future sustainability, which could lead to some alignment between the two themes. To make this alignment clearer, we added the following clarification to the sentence (page 22)

*In line with the findings described in the first theme, members recognized that the strong leadership and mentorship demonstrated within the CDPP required time and effort and that the sustainability of this level of leadership and mentorship is challenging.*

The theme was presented through a description of the resources required for capacity building, “1) human resources, 2) money and time, and 3) training” (p. 22, line 427). From this list, items 1 and 2 are the least clear to me (I think that training is effectively described at the bottom of page 23). Specifically, what is meant by “human resources”? Are “human resources" created through inputs of money or time as I think is suggested by the quote from Oliver? Is money and time one concept in which money can be converted to time and time to money? If not (as again suggested by Oliver, possibly) what are the conditions that sometimes allow “a little bit of extra money” to buy the time that is needed as compared to those where “a little bit of extra money…doesn’t really help”?

Thank you for pointing this out. Human resources refer to the people within the CDPP network who actively contribute to the development and sustainability of the network. Participants emphasized the need for dedicated staff or personnel to connect and liaise across research groups, coordinate projects, and serve in roles such as liaisons or coordinators. They also noted that human resources are essential for fostering and maintaining relationships between researchers and research users (e.g., community organizations, policy makers), and for facilitating meaningful human connection. This aligns closely with the principled approach outlined in the previous theme, particularly the principle that human connection is the foundation of meaningful and respectful partnerships.

Regarding the question about whether human resources are created through inputs of time and money: yes, participants suggested that human resources are supported by these inputs. In particular, they referred to the additional time and funding required to enable research users to participate meaningfully and in ways that are beneficial to them. This also connects to the earlier theme of mutual benefit.

Participants also indicated that money is needed to compensate research users for their time. However, they also emphasized that a flexible approach is often necessary, as money alone does not always guarantee successful partnerships. Oliver’s quote illustrates this well: while money can sometimes help by freeing up time or enabling participation, in other cases, it may not address the underlying needs. This highlights the importance of tailoring support to each partnership and having open conversations about what is needed. These insights also align with the principles of transparent communication, mutual benefit, as discussed in the previous theme.

To address this comment, we provided more details on the description of this theme. The revised paragraph reads now as follows (page 23/24)

*Human resources refer to the people within the CDPP network who actively contribute to the development and sustainability of the network. Participants recognized that human resources are an area of capacity building that the CDPP can leverage moving forward. They emphasized the need for dedicated staff or personnel to connect and liaise across research groups, coordinate projects, and serve in roles, such as liaisons or coordinators. Human resources are also essential for fostering and maintaining relationships between researchers and research users (e.g., community organizations, policy makers), and for facilitating meaningful human connection, an aspect that aligns closely with the principled approach outlined in the previous theme. Beyond human resources, dedicated time and money were also identified as critical to sustain the network and partnerships. Both researchers and research users recognized that money for partnership is imperative; without fair compensation, partnership work cannot take place. Financial resources (i.e., money) is needed to compensate research users for their time and to support engagement activities to bring people together. However, participants emphasized that money alone is not always sufficient to ensure successful partnership. Research users also need other types of support, such as protected time to engage in the research partnerships. They explained that money is helpful; however, without the ability to designate time outside of their current responsibilities, engaging in a research partnership is difficult:*

*“A lot of our community organizations are already maxed out in terms of our work and so to take on additional workload, a little bit more money to pay one of my staff doesn't really help. Sometimes we need to bring in extra people to either backfill a position or to do the actual knowledge translation work. So, sometimes it's just about, a little bit of extra money and sometimes it's about just providing other types of time and resources.” (Oliver, research user)*

*As illustrated by Oliver, a flexible and tailored approach is essential for successfully supporting and sustaining partnerships over the long term. Each partnership within the network may need something different, and having open conversations about the type of support needed is crucial for moving forward, which also aligns with the principles of transparent communication, mutual benefit, as discussed in the previous theme.*

In reading the authors’ description of this theme, I was yearning for clarification about each of the listed items and how they fit together. Most notably, I had the sense that this is not a list of three items (or maybe four), but instead some sort of triad where the items interact to produce the capacity that is necessary for leadership. Of course, my sense of what these concepts are and how they fit together is somewhat speculative – based upon the minimal data and analysis that were presented. A return to the interview transcripts would be necessary to effectively flesh out the participants’ views related to network maintenance.

The reviewer is correct in noting that human resources, money and time, and training are presented as distinct aspects, yet they are closely related and complementary. All three are essential for sustaining the CDPP network and its partnerships.

Human resources depend on financial support and time to function effectively over the long term. In turn, money and time enable the provision of training, which enhances the capacity of human resources and strengthens collaboration. Training also helps ensure that time and financial investments are used effectively, as well-equipped members are more likely to engage in meaningful and productive partnerships.

At the network level, all three components are necessary for long-term sustainability. At the partnership level, as illustrated by Oliver, support should be flexible and tailored to the specific needs of each individual partnership. A principled approach, guided by values such as mutual benefit, transparent communication, and human connection, can help foster meaningful and respectful collaborations.

To address this comment, we have added the following paragraph at the end of the results section to explain how these three aspects relate to each other (page 24)

*While human resources, money and time, and training are listed as distinct aspects, they are closely interconnected and mutually reinforcing. For example, human resources require financial support for long-term sustainability. Similarly, money and time are essential to provide training opportunities, which then enhance the capacity of human resources. Training plays a key role in building the skills and knowledge needed for meaningful partnerships, ensuring that members are better equipped to work together. Training also helps maximize the impact of the time and money invested, as members who are trained in partnership may be more likely to engage in meaningful and productive partnerships.*

*At the network level, all three aspects are necessary for long-term sustainability. At the partnership level, however, the type of support may need to be tailored and flexible, depending on the specific needs of each partnership. Aligning with the previous theme, a principled approach, guided by principles as mutual benefit, transparent communication, and human connection, can help foster meaningful and respectful partnerships.*

Should the authors enhance this theme, I would equally suggest that they also review the last part of the Transformational leadership and mentorship sub-section of the Discussion.

Thank you for pointing that out. We added the following sentence to the Discussion section to highlight the tailored approach.

*Such strategies require different types of resources (human, time, money, training), in which a tailored approach may be needed to best support individual partnerships within the network.*

6. PLOS authors have the option to publish the peer review history of their article ([what does this mean?](https://track.editorialmanager.com/CL0/https:%2F%2Fjournals.plos.org%2Fplosone%2Fs%2Feditorial-and-peer-review-process%23loc-peer-review-history/1/010f01978244eb8b-ff35a352-807b-4b84-8e2e-788e8f31a3ca-000000/pkTj4P-HvCdqp8lDixrKCqxrFNTci0973SpkmodLhnE=216)). If published, this will include your full peer review and any attached files.

If you choose “no”, your identity will remain anonymous but your review may still be made public.

**Do you want your identity to be public for this peer review?** For information about this choice, including consent withdrawal, please see our [Privacy Policy](https://track.editorialmanager.com/CL0/https:%2F%2Fwww.plos.org%2Fprivacy-policy/1/010f01978244eb8b-ff35a352-807b-4b84-8e2e-788e8f31a3ca-000000/GBKK1AgOVonUyb36a3YSAC2f24CHp4_9jBFRDikJFoY=216).

Reviewer #1: No

Reviewer #2: **Yes:** Shaun Cleaver
